# The Relationship between Capital Domains and Resilience in Facing the COVID-19 Pandemic in Indonesia

**DOI:** 10.4314/ejhs.v33i3.2

**Published:** 2023-05

**Authors:** Arief Hargono, Febi Dwirahmadi, Kurnia Dwi Artanti, Erni Astutik, Siti Shofiya Novita Sari, Febriyanti

**Affiliations:** 1 Department of Epidemiology, Biostatistics, Population Studies and Health Promotion, Faculty of Public Health, Universitas Airlangga, Surabaya, Indonesia; 2 Centre for Environment and Population Health, School of Medicine, Griffith University, Queensland, Australia; 3 Global Fund ATM, North Maluku Province, Indonesia; 4 Disaster Management Research Group, Post Graduate School of Airlangga University, Universitas Airlangga, Surabaya, Indonesia

**Keywords:** Capital domain, COVID-19, Economic capital, Physical capital, Resilience, Indonesia

## Abstract

**Background:**

Strengthening disaster resilience is important to protect existing development and in anticipation of various disasters and risks due to disasters such as the COVID-19 pandemic. This study aims to determine resilience among individuals in dealing with the COVID-19 pandemic in Indonesia based on the capital domains.

**Methods:**

This study used a cross-sectional design with 97 Indonesian people and was conducted using an online survey in May-December 2020. Data were analysed using multivariable logistic regression.

**Result:**

The results showed that 45.36% of the respondents had low resilience. Respondents whose expenses increased had 6.36 times higher odds of good resilience compared to respondents whose expenses decreased (AOR=6.36,95%CI=1.26–32,p=0.025). Respondents whose expenses were not affected had 12.32 times higher odds of having good resilience than respondents whose expenses were reduced (AOR=12.32,95%CI=1.82–83.40, p=0.01). Respondents with larger families had 32% lower odds of having good resilience than those with fewer family members (AOR=0.68, 95%CI=0.47–0.98, p=0.038). Respondents with no quarantine facilities had 65% lower odds of good resilience than those with quarantine facilities (AOR=0.35, 95%CI 0.13–0.95, p=0.04).

**Conclusion:**

Economic and physical capital as the part of capital domains showed a significant association with resilience during COVID-19 pandemics. Economic capital variables that had association with resilience were money expenses and the number of family members in household. Physical capital had a relationship with resilience were the availability of quarantine facilities. Government could encourage cooperation within the community to share economic resources. Local government could provide isolation facilities in local area.

## Introduction

The President of the Republic of Indonesia in 2020 officially declared corona virus disease (COVID-19) a national disaster. This determination was stated through the Presidential Decree Regarding the Stipulation of Non-Natural Disaster of the Coronavirus Disease 2019 (COVID-19) as a National Disaster. COVID-19 is an infectious disease that had already been declared a pandemic under the World Health Organization statement.

The COVID-19 pandemic brought various problems to Indonesian communities and created a unique context of ongoing disasters with severe impacts on daily life, including uncertainty, the lack of a clear timeline for its ending, the risk of death, increasing stressors and a lack of access to self-protection measures. Furthermore, the increasing number of COVID-19 cases had an impact on social and economic factors that greatly affected people's lives. The COVID-19 pandemic had a major impact on agriculture and food supply chains, which resulted in food insecurity in certain communities (1). The tourism sector also experienced an impact due to COVID-19 (2). Communities lacking good resilience faced exacerbated conditions such as mental health problems (3). Furthermore, the COVID-19 pandemic also disrupted the healthcare system (4).

Of all the impacts of COVID-19, the individual's ability to survive or maintain short- and long-term resilience in the face of the pandemic was the paramount importance(5). Individuals and family members of COVID-19 patients were at risk of experiencing psychological trauma (6). As described by Lokosang, resilience is both the process and outcome of adapting to difficult circumstances (7). The consequent need for independence made the development of resilience a crucial risk management goal (8). As a result, resilient people could withstand the shock and rebuild their conditions after the pandemic (9).

The capital domain is an important framework for measuring disaster resilience. It includes social, economic, physical, human, and natural factors. These five areas are strategic components to support sustainable development and poverty alleviation programmes(9,10). A study has shown that there is a relationship between factors affecting human capital and resilience in facing the COVID-19 pandemic (2). In addition, social capital measures, such as trust in other people, community membership, interacting with friends and availability of facilities, were also closely related to characteristics of resilience(11). Other research also showed that income as part of the economic capital was an important factor in influencing resilience in the face of COVID-19 (12).

Strengthening disaster resilience was important to protect existing development and to anticipate, prevent, adapt and reduce various shocks, pressures, risks and uncertainties due to disasters. There has been previous research on disaster resilience in the context of natural disasters (13). However, little was known about disaster resilience to the COVID-19 pandemic in Indonesia. Therefore, this research aims to examine issues of the capital domains that are related to resilience in dealing with the COVID-19 pandemic in Indonesia.

## Material and Methods

**Design of study**: The design of this study was cross-sectional, using quantitative data. Data was collected through online survey platforms (i.e. Kobo Toolbox) and social media (i.e. WhatsApp and Facebook). Since data collection was carried out during a pandemic, it was conducted online. Therefore, the possibility of information and selection bias was a limitation of this study. However, to minimise this bias, online data collection methods were regulated through the following provisions:

1. Respondents read the Pre-Research Explanation form, which contained information about the inclusion and exclusion criteria.
2. If the respondent agreed to participate in the research, they were required to sign the Informed Consent Form.
3. The questionnaire was designed to be completed only once by the respondent, identified by the informed consent form and email address.

Respondents were healthcare workers, patients, patients' families, and community members who were accessible and willing to fill out this survey. The research was located in Indonesia and carried out in May-December 2020. This study passed the ethical test of the Ethics Committee of the Faculty of Public Health, Universitas Airlangga No.99/EA/KEPK/2020.

**Outcome variable**: The outcome variable of the study was the resilience variable. Resilience was measured by the 10-item Connor-Davidson Resilience Scale (14). This instrument consisted of 10 question items as follows: 1) I am able to adapt to change; 2) I can deal with whatever comes; 3) I try to see the humorous side of problems; 4) Coping with stress can strengthen me; 5) I tend to bounce back after illness or hardship; 6) I can achieve goals despite obstacles; 7) I can stay focused under pressure; 8) I am not easily discouraged by failure; 9) I think of myself as a strong person; 10) I can handle unpleasant feelings. A 5-point Likert scale was used to provide possible answers that best aligned with the respondents' view to measure their level of resilience, i.e. strongly disagree = 1, disagree = 2, sometimes = 3, agree = 4, and strongly agree = 5. Responses were summed to produce a total score. The total score was categorised based on the median value (data not normally distributed), i.e. if the score was <40, then resilience was considered low, and if the score was 40 and above, then resilience was considered high.

**Explanatory variables**: The explanatory variables were capital domains that consisted of human, economic, physical, social and natural capital, as defined by Mayunga's 2007 study. Mayunga measured five capital domains (i.e. human, economic, physical, social and natural capital)(10).

Human capital was measured according to educational level (high school, college/university), employment status (not employed, employed), knowledge of COVID-19 (low and high), and attitude towards COVID-19 (negative, positive) variables

Economic capital was measured according to responses on the impact of the COVID-19 pandemic on income (increased, decreased, not impacted); expenses or expenditures (increased, decreased, not impacted); time to cover the cost of living (less than three months, more than four months); saving capabilities to cover monthly expenses (I cannot save at all, I can sometimes save but use it right away, I can save regularly); insurance ownership (no, yes);the number of family members in the household; and the number of working family members.

Natural capital was measured by the type of residence (urban, rural); availability of natural resources to meet daily needs (no, yes); and availability of natural resources in the environment to meet daily needs (no, yes). Physical capital was measured by the availability of health services (no, do not know, yes); availability of quarantine facilities (no, yes); and availability of personal protective equipment (available, unavailable).

Social capital was measured by trust in the government (no, yes); perception of cultural norms (bad, good); availability of task force team (available and unavailable); and perception of government assistance (disagree, agree). Respondents were further asked to provide information regarding their personal characteristics, including their status (patient's family member, community member, medical personnel); sex (man or woman); and age.

**Data analysis**: This study used a multivariable logistic regression model to answer its research objective. The data were analysed using STATA 14.2. The relationship between independent and control variables with resilience reported in the bivariable analysis was then examined using multiple logistic regression to control potential confounding variables. All variables with a p-value <0.25 in the bivariable analysis were included in the initial multivariable model. A variable was considered confounding when it was excluded in a multivariable model, and the other variables changed by 10% for the estimated Odds Ratio (OR). All confounding was stored in the final multivariable model. A confounding selection was carried out using the backward elimination method.

## Results

**Characteristics of respondents**: There were 139 respondents participating in this study. However, only 97 respondents had complete information on the outcome variable and the independent variable, and incomplete data were excluded from the analysis. As shown in Table 1, 45.36% of respondents were considered to have low resilience in facing the COVID-19 pandemic. Most of the respondents were medical practitioners (52.58%). The majority of the respondents were female (63.92%). The mean age of respondents was 33.35 years.

**Table 1:** Distribution of Characteristics of Respondents and Resilience Status during the COVID-19 Pandemic

Variables	N (%)	Mean (±SD)
Resilience status		
Low	44 (45.4)	
High	53 (54.6)	
Respondent status		
Patient's family	9 (9.3)	
Community	37 (38.1)	
Medical personnel	51 (52.6)	
Sex		
Male	35 (36.1)	
Female	62 (63.9)	
Age (years)		33.35 (9.79)

**Human capital**: The majority of respondents had finished college/university (94.85%) and were employed (88.66%). In addition, 70.1% of respondents had significant knowledge of COVID-19, and 67.01% of respondents had a positive attitude towards COVID-19 (Table 2).

**Table 2:** Distribution of Capital Domains Respondents during the COVID-19 Pandemic

	Variables	N	%	Mean	SD
**Human Capital**	Education level				
	High school	5	5.15		
	College/university	92	94.85		
	Employment status				
	Not employed	11	11.34		
	Employed	86	88.66		
	Knowledge of COVID-19				
	Low	29	29.9		
	High	68	70.1		
	Attitude towards COVID-19				
	Negative	32	32.99		
	Positive	65	67.01		
**Economic Capital**	COVID-19 pandemic impact on income				
	Decreased	34	35.05		
	Increased	6	6.19		
	Not impacted	57	58.76		
	COVID-19 pandemic impact on expenditure				
	Decreased	15	15.46		
	Increased	64	65.98		
	Not impacted	18	18.56		
	Time to cover the cost of living				
	Less than 3 months	48	49.48		
	More than 4 months	49	50.52		
	Savings capability to cover monthly expenses during the COVID-19 pandemic				
	I cannot save at all	17	17.53		
	I can sometimes save, but use it right away	45	46.39		
	I can save regularly	35	36.08		
	Insurance ownership				
	No	19	19.59		
	Yes	78	80.41		
	Number of family members in the household			3.825	1.53
	Number of working family members			1.887	0.95
**Physical Capital**	Availability of health services				
	No	5	5.15		
	Do not know	4	4.12		
	Yes	88	90.72		
	Availability of quarantine facilities				
	None	45	46.39		
	Exist	52	53.61		
	Availability of personal protective equipment				
	Uncompleted	76	78.35		
	Completed	21	21.65		
**Social Capital**	Trust in the government				
	No	15	15.46		
	Yes	82	84.54		
	Perception of government assistance				
	Disagree	27	27.84		
	Agree	70	72.16		
	Perception of cultural norms				
	Bad	47	48.45		
	Good	50	51.55		
	Availability of task force team Available	74	76.29		
	Unavailable	23	23.71		
**Natural Capital**	Type of residence				
	Rural	31	31.96		
	Urban	66	68.04		
	Availability of natural resources to meet daily needs				
	Not	73	75.26		
	Yes	24	24.74		
	Availability of natural resources in the environment to meet daily needs				
	Not	64	65.98		
	Yes	33	34.02		

**Economic capital**: Table 1 shows that 58.76% had not experienced any change in their income during COVID-19. However, the pandemic affected respondents' expenditures, as 65.98% of the respondents were spending significantly more. 50.52% of respondents stated that their savings were able to cover their expenses for more than four months; 46.39% of the respondents said that they could save, but then their savings were used immediately. Most of the respondents (80.41%) had health insurance (Table 2).

**Physical capital**: Most of the respondents (90.72%) were aware of available health services. 46.39% of respondents did not have quarantine facilities, and 78.35% of the respondents stated that they did not have complete personal protective equipment (Table 2).

**Social capital**: The majority of respondents (84.54%) trusted the government, and 72.16% of respondents supported the government's policies of providing incentives and assistance. There were 51.55% of respondents with a good perception of cultural norms. Most of the respondents (76.29%) also stated that there was a task force team in their neighbourhood (Table 2).

**Natural capital**: Most respondents lived in urban areas (68.04%). As many as 73 people (75.26%) stated that they did not have land that could be used for daily needs, and 65.98% of respondents also emphasised that they did not have land in the neighbourhood that could meet their daily needs (Table 2).

Variables that had a p-value of less than 0.25 in bivariable analysis were included in the initial multivariable model, namely, the impacts of the pandemic on income and expenditures; the number of family members in a household; the availability of health services; the availability of self-isolation rooms; the availability of personal protective equipment; trust in the government; and the location of a respondent's residence. Human capital (employed status, knowledge of COVID-19, attitude towards COVID-19), natural capital (type of residence, availability of natural resources, personal protective equipment), and social capital (trust in the government, perception of cultural norms, government assistance, availability of a task force team) did not have an association with resilience in facing the COVID-19 pandemic. However, economic capital and physical capital did have an association with resilience in facing the COVID-19 pandemic (Table 3).

**Table 3:** Bivariable and Multivariable Analysis of Community Resilience during the COVID-19 Pandemic

Variables	Bivariable				Multivariable			

	OR	95% CI		p-value	AOR*	95% CI		p-value
		Lower	Upper			Lower	Upper	
**Human Capital**								
Employment status								
Not employed	Ref							
Employed	0.66	0.18	2.41	0.527				
Knowledge of COVID-19								
Low	Ref							
High	0.97	0.4	2.32	0.945				
Attitude towards COVID-19								
Bad	Ref							
Good	1.6	0.68	3.74	0.283				
**Economic Capital**								
COVID-19 pandemic impact on income								
Decreased	Ref				Ref			
Increased	0.14	0.01	1.33	0.087	0.14	0.01	1.78	0.13
Not impacted	0.89	0.38	2.12	0.802	0.61	0.19	1.95	0.401
The COVID-19 pandemic's impact on money expense								
Decreased	Ref				Ref			
Increased	3.77	1.08	13.12	0.037	6.36	1.26	32.03	0.025
Not impacted	5.49	1.22	24.81	0.027	12.32	1.82	83.4	0.01
Time to cover the cost of living								
Less than 3 months	Ref							
More than 4 months	1.23	0.55	2.73	0.617				
Savings capability to cover monthly expenses								
I cannot save at all	Ref							
I can sometimes save but use it right away	0.62	0.19	1.98	0.422				
I can save regularly	0.58	0.17	1.91	0.368				
Insurance ownership								
No	Ref							
Yes	0.85	0.31	2.34	0.751				
Number of family members in the household	0.77	0.585	1.02	0.072	0.68	0.47	0.98	0.038
Number of working family members	0.87	0.57	1.33	0.518				
**Natural Capital**								
Type of residence								
Rural	Ref				Ref			
Urban	1.75	0.74	4.15	0.201	2.1	0.71	6.23	0.181
Availability of natural resources to meet daily needs								
Not	Ref							
Yes	1.54	0.59	3.95	0.374				
Availability of natural resources in the environment to meet daily needs								
Not	Ref							
Yes	0.99	0.43	2.31	0.989				
**Physical Capital**								
Availability of health services								
Not		Ref				Ref		
Do not know	0.08	0.01	1.95	0.122	0.06	0	10.86	0.295
Yes	0.3	0.03	2.79	0.29	0.61	0.04	9.59	0.723
Availability of quarantine facilities								
Exist	Ref				Ref			
No	0.55	0.24	1.23	0.144	0.35	0.13	0.95	0.04
Availability of personal protective equipment (PPE)								
Incomplete	Ref				Ref			
Complete	0.55	0.21	1.45	0.224	0.76	0.22	2.61	0.664
**Social Capital**								
Trust in the government								
No	Ref				Ref			
Yes	0.38	0.11	1.29	0.123	0.3	0.08	1.22	0.093
Perception of cultural norms								
Bad	Ref							
Good	1.12	0.5	2.49	0.781				
Availability of task force team								
Available	Ref							
Unavailable	1.39	0.54	3.62	0.493				
Perception of government assistance								
Disagree	Ref							
Agree	0.95	0.39	2.32	0.91				

Results showed that respondents whose expenses increased had 6.36 times higher odds of having good resilience than respondents whose expenses decreased after being controlled by other variables (AOR=6.36, 95%CI 1.26–32, p=0.025). In addition, respondents whose expenses were not affected had 12.32 times higher odds of having good resilience than respondents whose expenses were reduced after being controlled by other variables (AOR=12.32, 95%CI 1.82–83.40, p=0.01) (Table 3).

Respondents with larger families had32% lower odds of having good resilience than respondents with fewer family members after being controlled by other variables (AOR=0.68, 95%CI=0.47–0.98, p=0.038). Furthermore, respondents who did not have quarantine facilities had 65% lower odds of having good resilience than respondents who had proper quarantine facilities after being controlled by other variables (AOR=0.35, 95%CI 0.13–0.95, p=0.04) (Table 3).

## Discussion

**Characteristics of respondents**: The results of the study showed that the respondents' characteristics, such as status, sex and age, did not have an association with resilience. A study also found that age was not related to stress, even though older people were more resilient than younger people when dealing with COVID-19 (15). In addition, a study conducted in China found that there are differences between men and women in dealing with COVID-19 which women were more at risk of having a lower level of resilience compared to men(16). Our results showed that almost half of the respondents had low resilience in dealing with the COVID-19 pandemic. The results of other studies found that people have low resilience when facing COVID-19 (17).

**Human capital**: Human capital, which included employment status, knowledge status and attitude variables, did not have an association with resilience during the COVID-19 pandemic. The results of this study did not align with research conducted in Latin American countries(18). A study found that intervention in knowledge and perceived knowledge was closely related to resilience (19). The level of employment also affected the level of resilience(20). This difference in results might be due to the fact that COVID-19 is a new disease and confusing information appeared in the community causing high levels of public panic(21). At the time of the pandemic, a policy on restriction was implemented nationally. The policy was in the form of government regulations regarding large-scale social restrictions to handle COVID-19 (22). It caused everyone to have the same attitude and concern during the COVID-19 pandemic.

**Economic capital**: Economic capital could be measured by the COVID-19 pandemic's impact on expenditures. This study showed that respondents whose expenses increased had higher odds of having good resilience than respondents whose expenses decreased after being controlled by other variables. According to research conducted by Zhong in terms of financial capital, the Chinese population with a relatively high socioeconomic status had good knowledge, optimism, and proper practices towards COVID-19 during the beginning of the increase in COVID-19 cases (23). Nonetheless, the economic shocks from the COVID-19 pandemic are most likely much greater than those seen since the 2008–2009 financial crisis. The spread of COVID-19 has indicated high human costs, and with public health systems struggling to cope, these costs will continue to rise. The COVID-19 pandemic has resulted in economic consequences of adverse health shocks in households. The household incomes of many families are likely to decrease as the unemployment rate increases. In many households, especially poor households, this decrease in household income will also reduce their investment in education. It will be worsened by the health shocks associated with the pandemic (24).

This study also showed that respondents whose expenses were not affected during the COVID-19 pandemic had significantly higher resilience than respondents with reduced expenditures during the COVID-19 pandemic. This result aligns with research conducted by Martin in the San Francisco Bay Area, showing that household expenditures fell significantly, and it took nearly a year on average for individuals to recover (25). The long recovery time after the crisis could be further exacerbated by falling demand, changes in people's consumption behaviour and a general slowdown in economic activity. In addition, this may be because household socioeconomics is related to income. Poor households are less resilient and more likely to fall into poverty due to COVID-19 (26). Other study has also stated that during the COVID-19 pandemic, especially during large-scale restrictions, there were severe economic losses for industry and disruptions to companies (27).

This study showed that respondents with more family members had a worse resilience rate than those with fewer family members. As the smallest unit in society that is considered the first environment for children and the main environment for family members, the family has an important role in making the community more capable of preventing COVID-19 (28). Research conducted by Ainuddin and Routray that compared two regions, i.e. Zone A and Zone B, showed that a high proportion of dual-income sources greatly helped the community be more resilient and restored after the disaster (29). It may be because during the COVID-19 pandemic, most people reported losing their jobs, so respondents with larger families would face a higher financial burden.. Other evidence suggested that pandemics could exacerbate inequality if powerful groups used unavailable resources for personal gain (30). The result of the research in North Maluku, Indonesia, showed that economic factors were an important component of resilience, including house ownership and income, especially for communities with more than one source of income (31).

**Physical capital**: This research found that respondents who did not have proper quarantine facilities had lower odds of good resilience than respondents with appropriate quarantine facilities. The availability and accessibility of quarantine facilities as physical capital were considered to play a major role in making the community more resilient to the COVID-19 pandemic. For people infected with COVID-19, it is mandatory to self-isolate to prevent further infection to others. Isolated communities will continue to get medical help needed and stay in touch with doctors because the severity of the virus can be fatal and ensure help and support when respiratory distress or other emergency warning signs are found (32). The community must also continue personal care and treatment as appropriate so that the body remains healthy and healed (33).

Healthcare facilities must identify the services that can be provided and the priority of services, taking into account the benefits and risks of responding with limited resources during the COVID-19 pandemic and reducing exposure and transmission risks for the community itself and health or non-health workers in healthcare facilities. In addition, it is also known that some types of PPE are required to prevent the transmission of COVID-19, namely masks, face shields and gloves when necessary. The result of this study showed that there were no associations between the availability of healthcare services and personal protective equipment. The results of this study may be due to the use of PPE suggested by the government and the World Health Organization (WHO). In addition, during the pandemic, people were afraid to come to health services for fear of being diagnosed with COVID-19 and infected with the disease.

This study showed that respondents with proper quarantine facilities had better resilience than respondents who did not have appropriate quarantine facilities (e.g. self-isolation rooms). Isolation or quarantine can be conducted independently in individual homes or places determined by the government while coordinating with regional health centre officials. Regarding social capital, Ferreira found that study respondents whose resilience decreased reported that they needed greater help and cooperation from others (e.g. family, friends or neighbours) to recover from the impact of COVID-19 (5).

**Natural capital**: Natural capital describes natural resource reserves that are useful for providing and supporting living well-being and livelihoods or natural resources available to individuals and communities (34). The natural characteristics of urban and rural areas are different. This difference also causes economic differences between the people who live in rural and urban areas. The results of our study indicate that although the characteristics of rural and urban communities are different, these differences do not cause differences in the status of resilience in the community. COVID-19 is a new disease that caused a global public health concern and emergency. This disease was a concern for everyone, both in rural and urban areas. Therefore, all people had the same feelings of panic (35). According to Carter and Cordero (2022), the majority of people responding to the COVID-19 crisis were able to move forward with positive attitudes, learned to live with uncertainty, relied on creativity to solve problems and became more confident in their ability to solve problems due to the outcome of the pandemic (36). Therefore, all levels of society, both urban and rural, were the same in terms of facing the pandemic crisis. During the pandemic, the government provided social assistance to overcome the socioeconomic impact of the COVID-19 pandemic. This condition was expected to ease the burden on society in urban and rural areas (37).

**Social capital**: Social capital has an important role in the community. It is often a critical factor in resilience to disasters. High levels of social capital also often coincide with manifestations of trust, reciprocity, collective action, information sharing and participation in societal activities. All these things are valuable for developing and implementing disaster risk reduction efforts that are beneficial for building resilience in the community. Community resources and relationships can provide a platform for disaster risk reduction(38). However, our study found no association between trust in the government and community resilience. During the COVID-19 pandemic, there was a lot of information circulating about the virus. The information circulating came from various sources that were not all true, such as hoaxes, personal opinions and conspiracy theories(39).

**Strengths and weaknesses of the study**: The strength of this study is that the variables used to measure the risk factors of resilience status in the community use capital domains, namely, human, economic, natural, physical, and social capital. Research on resilience to disasters or pandemics/endemics based on domain capital has not been widely carried out. A study related to the capital domain also did not all measure the levels of resilience regarding all capital domains (economic, natural, human, social and physical)(40). The limitation of this research is that it was conducted during the COVID-19 pandemic, so the data collection was online. The limited data was obtained from the respondents who received the online questionnaire link. In addition, 139 respondents participated in this study, but only 97 gave complete information.

In conclusion, he capital domains showing a significant association with resilience were economic and physical capital. Economic capital that had a relationship with resilience was the COVID-19 pandemic's impact on money expenses and the number of family members in a household. Physical capital that had a relationship with resilience was the availability of quarantine facilities. The results of this study lead to policy recommendations in which the government could encourage mutual cooperation among community members to share economic resources to help each other. Households in the community could share their contributions, such as money, food, material and medicine. Households could provide a specific isolation room to minimise contact with other family members. Furthermore, local governments could provide isolation facilities in their local area.
